# All-*Trans* Retinoic Acid Induces TGF-β_2_ in Intestinal Epithelial Cells via RhoA- and p38α MAPK-Mediated Activation of the Transcription Factor ATF2

**DOI:** 10.1371/journal.pone.0134003

**Published:** 2015-07-30

**Authors:** Kopperuncholan Namachivayam, Krishnan MohanKumar, Dima Arbach, Ramasamy Jagadeeswaran, Sunil K. Jain, Viswanathan Natarajan, Dolly Mehta, Robert P. Jankov, Akhil Maheshwari

**Affiliations:** 1 Department of Pediatrics, University of Illinois at Chicago, Chicago, Illinois, United States of America; 2 Department of Pediatrics, Morsani College of Medicine, University of South Florida, Tampa, Florida, United States of America; 3 Department of Pediatrics, University of Texas Medical Branch, Galveston, Texas, United States of America; 4 Department of Pharmacology, University of Illinois at Chicago, Chicago, Illinois, United States of America; 5 Physiology & Experimental Medicine Program, Hospital for Sick Children Research Institute, Toronto, Ontario, Canada; 6 Department of Physiology, University of Toronto, Toronto, Ontario, Canada; 7 Department of Pediatrics, University of Toronto, Toronto, Ontario, Canada; 8 Department of Molecular Medicine, Morsani College of Medicine, University of South Florida, Tampa, Florida, United States of America; 9 Department of Community and Family Health, College of Public Health, University of South Florida, Tampa, Florida, United States of America; Purdue University, UNITED STATES

## Abstract

**Objective:**

We have shown previously that preterm infants are at risk of necrotizing enterocolitis (NEC), an inflammatory bowel necrosis typically seen in infants born prior to 32 weeks’ gestation, because of the developmental deficiency of transforming growth factor (TGF)-β_2_ in the intestine. The present study was designed to investigate all-*trans* retinoic acid (atRA) as an inducer of TGF-β_2_ in intestinal epithelial cells (IECs) and to elucidate the involved signaling mechanisms.

**Methods:**

AtRA effects on intestinal epithelium were investigated using IEC6 cells. TGF-β_2_ expression was measured using reverse transcriptase-quantitative polymerase chain reaction (RT-qPCR) and Western blots. Signaling pathways were investigated using Western blots, transiently-transfected/transduced cells, kinase arrays, chromatin immunoprecipitation, and selective small molecule inhibitors.

**Results:**

AtRA-treatment of IEC6 cells selectively increased TGF-β_2_ mRNA and protein expression in a time- and dose-dependent fashion, and increased the activity of the TGF-β_2_ promoter. AtRA effects were mediated via RhoA GTPase, Rho-associated, coiled-coil-containing protein kinase 1 (ROCK1), p38α MAPK, and activating transcription factor (ATF)-2. AtRA increased phospho-ATF2 binding to the TGF-β_2_ promoter and increased histone H2B acetylation in the TGF-β_2_ nucleosome, which is typically associated with transcriptional activation.

**Conclusions:**

AtRA induces TGF-β_2_ expression in IECs via RhoA- and p38α MAPK-mediated activation of the transcription factor ATF2. Further studies are needed to investigate the role of atRA as a protective/therapeutic agent in gut mucosal inflammation.

## Introduction

In the developing intestine, transforming growth factor-beta 2 (TGF-β_2_) plays an important role in the maturation of epithelial cells and resident immune cells, and promotes normal development of mucosal tolerance to bacterial products in these cells [1–4]. We have previously shown that preterm infants are developmentally-deficient in intestinal TGF-β_2_ expression, which predisposes them to necrotizing enterocolitis (NEC)—an idiopathic, acquired inflammatory bowel necrosis seen almost exclusively in infants born prior to 32 weeks of gestation [1,2]. Although the etiology of NEC is complex and not well-elucidated, current evidence indicates that NEC occurs when altered/disrupted mucosal barrier allows bacteria normally present in the intestinal lumen to translocate into the subepithelial *lamina propria*, resulting in a damaging mucosal inflammatory response. In this pathophysiological model, the unique predisposition of premature infants to NEC can be explained because the intestinal epithelium and resident host defense cells of the innate immune system are yet to undergo normal TGF-β-mediated inflammatory downregulation that is characteristic of the adult intestine, and trigger an exaggerated inflammatory response when exposed to bacteria or bacterial products [1,5].

In the neonatal intestine, TGF-β bioactivity is derived from local expression in the epithelium and from orally-ingested maternal milk [1,3,6]. TGF-β expression in intestinal epithelial cells (IECs) normally follows an autocrine feed-forward loop, but this autocrine induction is dampened in the premature intestine due to increased expression of Smad7 [2]. This developmental deficiency of TGF-β in the neonatal intestine can become clinically-evident in clinical situations with limited supply of milk-borne TGF-β_2_, such as in growth-restricted infants receiving less TGF-β_2_ from maternal milk [7], or in infants fed infant formula, which does not contain much TGF-β_2_ [8]. In this context, strategies to augment TGF-β_2_ expression in the preterm intestine are of potential interest in developing novel methods to prevent/treat NEC.

Retinoic acid induces TGF-β_2_ in diverse cell lineages such as keratinocytes, epidermal cells, hair follicles, non-small cell lung carcinoma cells, and pancreatic ductal adenocarcinoma cells [9–12]. However, the effects of retinoic acid on TGF-β expression are not always predictable and may need to be specifically evaluated in each individual organ system. For instance, retinoic acid inhibits TGF-β_2_ expression in the developing heart and in pancreatic acini [13–15]. In pregnant rats, administration of retinoic acid resulted in decreased TGF-β_2_ expression in the fetal cerebral cortex during early gestation, followed by a rebound increase in late gestation [16]. In the developing intestine, the effects of retinoic acid on TGF-β_2_ expression are not known. Because IECs are a major cellular source of TGF-β_2_ in the intestine [1], the present study was designed to investigate whether all-*trans* retinoic acid (atRA) can induce TGF-β_2_ in IECs and to identify downstream signaling mediator(s).

## Materials and Methods

### Animals

Animal studies were performed at the University of Texas Medical Branch (UTMB), Galveston. The protocol was approved by the Institutional Animal Care and Use Committee at UTMB Health Research Services, and the studies were performed in strict accordance with the recommendations in the Guide for the Care and Use of Laboratory Animals of the National Institutes of Health. Rat pups were fed all-*trans* retinoic acid (atRA; 500 μg) mixed in peanut oil on postnatal days 3 through 7. After euthanasia with CO_2_ inhalation, the intestines were harvested for further studies.

### Intestinal epithelial cells and reagents

IEC6 rat neonatal IECs, HT29 cells, and T84 cells (all from ATCC, Manassas, VA) were cultured under standard conditions [17] and were treated with all-*trans* retinoic acid (atRA; Sigma, St. Louis, MO). All chemical inhibitors used in this study were purchased from Santa Cruz Biotechnology, Santa Cruz, CA. SB203580, a pyridinyl imidazole, inhibits p38 mitogen-activated protein kinase (MAPK); Wortmannin is a selective inhibitor of the phosphatidylinositide 3-kinases (PI3K); SP600125 is a selective inhibitor of the c-Jun N-terminal kinases (JNK); PD98059 is a selective, cell-permeable inhibitor of the mitogen-activated protein kinase kinase 1 (MEK1); and Y-27632 dihydrochloride inhibits the Rho-associated, coiled-coil-containing protein kinases (ROCK).

### Plasmids and viral vectors

Luciferease reporter plasmid carrying the TGF-β_2_ promoter was purchased from GeneCopoeia, Rockville, MD USA. Control (null) adenovirus and adenoviral vectors carrying the wild-type and dominant negative p38 MAPK sequences (Gene Transfer Vector Core, University of Iowa, Iowa City, IA), and plasmids (pcDNA3.1+) carrying the constitutively-active RhoA GTPase with the G14V mutation and the dominant negative RhoA with the T19N mutation have been described previously [18–20]. Plasmid (pcDNA3) construct carrying the MKK6-p38α fusion protein has been described previously [21] and was a kind gift of Dr. Guan Chen, Medical College of Wisconsin). IEC6 cells were transfected using the lipofectamine 2000 reagent (Invitrogen, Grand Island, NY) per manufacturer’s instructions, and were used 24h later (pre-determined optimum).

#### Reverse transcriptase-quantitative polymerase chain reaction (RT-qPCR)

TGF-β_1_, TGF-β_2_, and TGF-β_3_ expression was measured using standard SYBR green I-based RT-qPCR [22]. Primers were designed using the Beacon Design software (Bio-Rad, Hercules, CA); the primer sequences were: TGF-β_1_: forward: ATTCCTGGCGTTACCTTGG, reverse: CCTGTATTCCGTCTCCTTGG; TGF-β_2_: AGGATACAATGCTAACTTCTG, reverse: GTAGAGGATGGTCACTGG; TGF-β_3_: CGGACCTTCTCGTCTCTTC, reverse: ATGGAGTTCAGTGTGTCAGG. Data were normalized against glyceraldehyde 3-phosphate dehydrogenase and groups were compared by the 2^–ΔΔCT^ method.

### Antibodies

The following antibodies were used for Western blots, immunocytochemistry, and chromatin immunoprecipitation (ChIP): rabbit polyclonal anti-TGF-β_2_ IgG (sc-90; Santa Cruz), Rabbit polyclonal anti-p38α IgG (AF8691; R&D), mouse monoclonal phospho-p38 IgM (sc-7973; Santa Cruz), rabbit polyclonal MKK6 IgG catalog #9264; Cell Signaling), mouse monoclonal anti-RhoA IgG1 (sc-418; Santa Cruz), mouse monoclonal anti-ROCK1 IgG_1_ (catalog #611137; BD Transduction Laboratories), mouse monoclonal anti-cleaved ROCK1 IgG_1_ (sc-52953; Santa Cruz), mouse monoclonal anti-ROCK2 IgG1 (catalog #610624; BD), rabbit polyclonal anti-activating transcription factor (ATF2) IgG (sc-187; Santa Cruz), rabbit polyclonal anti-phospho-ATF2 Thr7 IgG (sc-7982; Santa Cruz), goat polyclonal anti-β-actin IgG (sc-1616; Santa Cruz); rabbit polyclonal anti-acetyl-histone H2A Lys5 (H2AK5) IgG (cell Signaling Technology, Danvers, MA; catalog #2576), rabbit polyclonal anti-acetyl-H2BK5 IgG (catalog #2574; Cell Signaling), rabbit monoclonal acetyl-H3K9 IgG (catalog #9649; Cell Signaling), and rabbit polyclonal acetyl-H4K8 IgG (catalog #2594; Cell Signaling), rabbit polyclonal phospho-MAP kinase-activated protein kinase 2 (phospho-MAPKAPK-2; Thr334; catalog #3041; Cell Signaling) goat polyclonal Smad2 IgG (catalog #sc-6200; Santa Cruz), mouse monoclonal Smad3 IgG_2a_ (catalog #sc-101154; Santa Cruz), goat polyclonal Smad4 IgG (catalog #sc-1909; Santa Cruz), goat polyclonal Smad7 IgG (catalog #sc-9183; Santa Cruz), rabbit polyclonal phospho-Smad2 (Thr220) IgG (catalog #sc-135644; Santa Cruz).

### RhoA/Rac1/Cdc42 activation assay

The active forms of RhoA, Rac1, and Cdc42 GTPases were detected using a commercially-available kit (RhoA/Rac1/Cdc42 activation combo kit, Cell Biolabs, San Diego, CA). In its active (GTP-bound) state, RhoA binds specifically to the Rho-binding domain (RBD) of Rhotekin, and Rac1 or Cdc42 bind the p21-binding domain (PBD) of p21-activated protein kinase (PAK). This assay utilizes Rhotekin RBD and PAK PBD agarose beads to specifically isolate and pull down the active form of Rho/Rac/Cdc42 from cell lysates. The precipitated GTP-bound RhoA, Rac1, or Cdc42 is then detected by Western blots using specific antibodies.

### ROCK activity assay

We used a commercially-available ROCK activity assay (Millipore, Billerica, MA). This colorimetric enzyme immunoassay detects ROCK-mediated phosphorylation (Thr696) of the target myosin phosphatase target subunit 1.

### Western blots

TGF-β_2_, p38α and phospho-p38, RhoA, ROCK1, cleaved ROCK1, ROCK2, ATF2 and phospho-ATF2, MKK6, phospho-MAPKAPK2, acetyl-H2AK5, acetyl-H2BK5, acetyl-H3K9, acetyl-H4K8, Smad2, Smad3, Smad4, Smad7, and phospho-Smad2 expression was measured in IEC6 cells by Western blots using established methods [23].

### MAPK antibody array

AtRA-induced MAPK activation was investigated using a commercially-available antibody array with a panel of 24 different kinases (Proteome Profiler phospho-MAPK array, R&D, Minneapolis, MN) per manufacturer’s instructions. Briefly, after mixing with a cocktail of biotinylated detection antibodies, cell lysates were incubated with the array membrane, and phosphorylated kinases were captured by spotting specific antibodies on a nitrocellulose membrane. The membrane was developed using streptavidin-horseradish peroxidase and chemiluminescence detection reagents similar to Western blots.

### Immunocytochemistry

Phospho-p38, cleaved ROCK1, and phospho-ATF2 were detected in IEC6 cells by immunocytochemistry. Cells grown on cover-slips in serum-free media (Dulbecco’s modified Eagle’s media) were treated with atRA 10 μM × 2h, washed, and then fixed with ice-cold methanol and acetone (1:1) × 10 min at -20°C, blocked (Superblock T20 blocking buffer, Thermo Scientific) × 30 min, and then incubated overnight at 4°C with primary antibody: mouse anti-phospho P38 (Tyr 182), mouse anti- ROCK1, or rabbit anti-phospho ATF2 (Thr 71) (all from Santa Cruz). Secondary staining was performed with Alexa Fluor 546-conjugated goat anti-mouse IgG and/or Alexa Fluor 488 conjugated chicken anti-rabbit IgG (Invitrogen) × 1h at room temperature. Nuclear staining was obtained with DAPI (Invitrogen). Fluorescence imaging was performed using a Zeiss LSM 710 confocal microscope.

### ChIP

Phospho-ATF2 binding to the TGF-β_2_ promoter and the acetylation status of histones H2A and H2B on the TGF-β_2_ nucleosome was measured using a commercially-available ChIP assay (Magnify Chromatin Immunoprecipitation system, Invitrogen, Grand Island, NY) per manufacturer's instructions. Briefly, cells were cross-linked with formaldehyde at 1% final concentration × 10 min and the reaction was then stopped by adding glycine to a final concentration of 1.25 M. Chromatin was sheared by sonication (8 times, 20 sec pulses), and cleared by centrifugation. Immunoprecipitation was performed using anti-ATF2, anti-phospho-ATF2, anti-acetyl-H2AK5, anti-acetyl-H2BK5, or an isotype control from a different species (mouse) provided in the kit. Precipitated DNA was reverse cross-linked using provided buffers and then amplified by real-time PCR using primers specific for TGF-β_2_ promoter (forward: CGGACCTTCTCGTCTCTTC and reverse ATGGAGTTCAGTGTGTCAGG). Data were expressed as percent input after normalization for background [24].

### Statistical methods

Parametric and non-parametric tests were applied using the Sigma Stat 3.1.1 software (Systat, Point Richmond, CA). For PCR data, crossing-threshold (ΔΔCT) values for genes with ≥ 2-fold change were compared by the Mann-Whitney *U* test. Number of samples is indicated in each figure legend. In all tests, *p*<0.05 was accepted as significant.

## Results

### AtRA induces TGF-β_2_ expression in IECs

**Fig 1:**
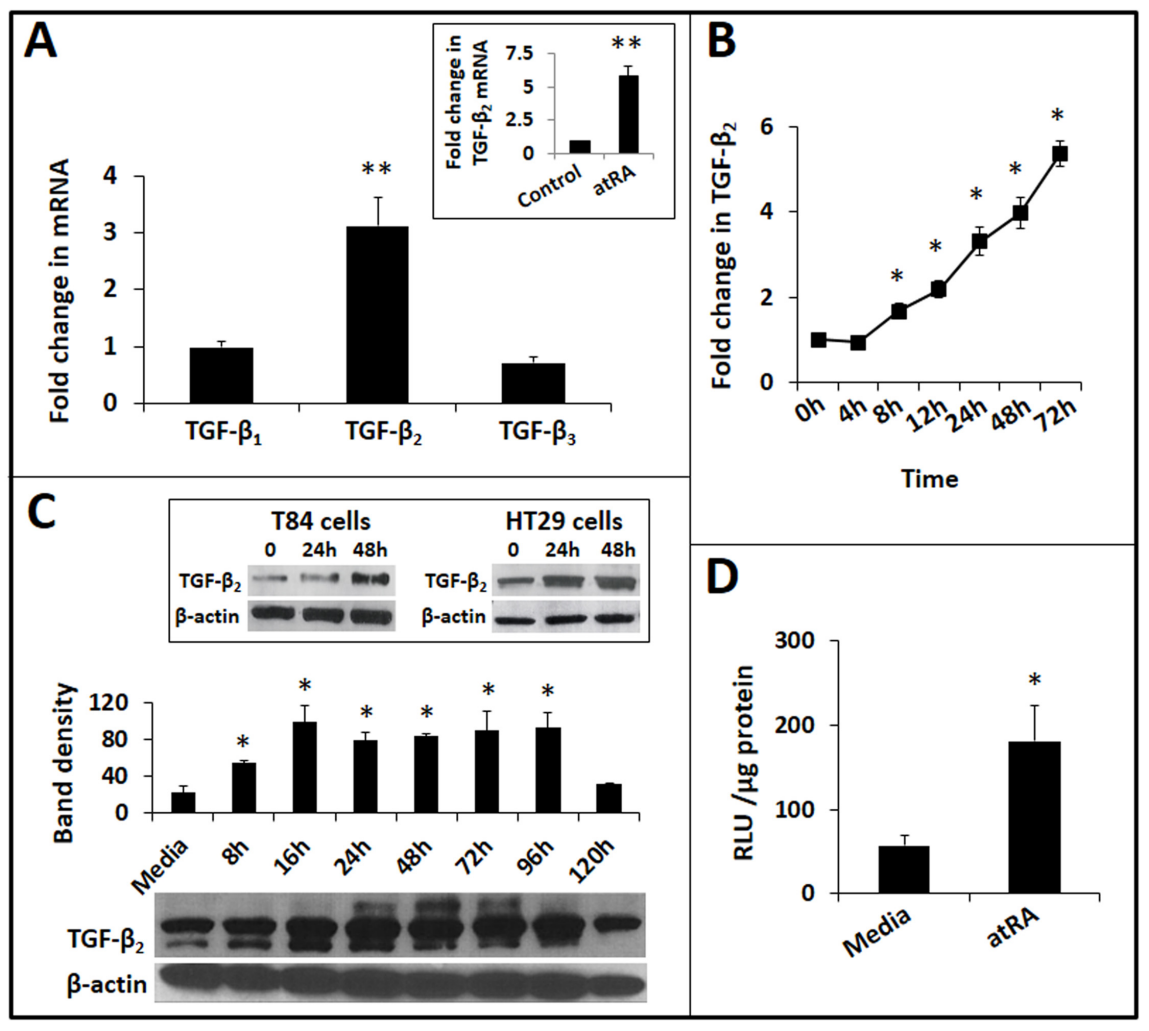
AtRA induces TGF-β_2_ expression in IECs. *A*. Bar-diagram (means ± SE) shows fold change in mRNA expression of TGF-β_1_, TGF-β_2_, and TGF-β_3_ in IEC6 cells treated with atRA (10 μM) × 24h. *Inset*: Rat pups receiving atRA orally show increased TGF-β_2_ expression in the small intestine on postnatal day 7. Bar-diagram (means ± SE) shows fold change in TGF-β_2_ mRNA. N = 5 pups per group. *B*. Temporal kinetics of atRA-induced TGF-β_2_ expression in IEC6 cells. Line diagram (means ± SE) shows fold changes in TGF-β_2_ mRNA expression over cells cultured in media alone, depicted as a function of the duration of atRA treatment. *C*. Western blots show atRA-induced TGF-β_2_ protein expression in IEC6 cells. Bar-diagram (means ± SE) summarizes densitometric data. *Inset*: Western blots showing increased TGF-β_2_ expression in atRA-treated T84 and HT29 human IECs, respectively. *D*. AtRA activates TGF-β_2_ promoter in IEC6 cells. Bar-diagram (means ± SE) shows relative luciferase activity in IEC6 cells transfected with a luciferase reporter containing the TGF-β_2_ promoter, 8h after treatment with ATRA. Data represent 3 separate experiments; * *p*<0.05, ** *p*<0.01.

To investigate retinoic acid effects on IECs, we treated IEC6 rat intestinal epithelial cells with atRA *in vitro*. IEC6 cells are primary IECs that were originally derived from duodenal crypt IECs from 18–24-day-old rat pups [25]. AtRA (1–10 μM) selectively induced the TGF-β_2_ isoform in IEC6 cells (**Fig 1A**). In support of these data, we detected a similar increase in TGF-β_2_ expression in the intestine in rat pups fed atRA (500 μg) mixed in peanut oil from postnatal day 3 through day 7 (***inset***).

AtRA treatment of IEC6 cells increased TGF-β_2_ mRNA expression starting at 8h and this response continued beyond 72h (**Fig 1B**). At the protein level, we detected increased TGF-β_2_ for up to 96h (**Fig 1C**). Further confirmation of atRA effects was obtained in HT29 and T84 human IECs, which showed a similar increase in TGF-β_2_ expression (***inset***). Finally, to confirm the mechanism of the observed induction of TGF-β_2_, IEC6 cells were transiently transfected to express a luciferase reporter driven by the TGF-β_2_ promoter. As shown in **Fig 1D**, atRA treatment increased the activity of the TGF-β_2_ promoter.

### AtRA-induced TGF-β_2_ expression in IECs is mediated via RhoA GTPase and ROCK1

**Fig 2:**
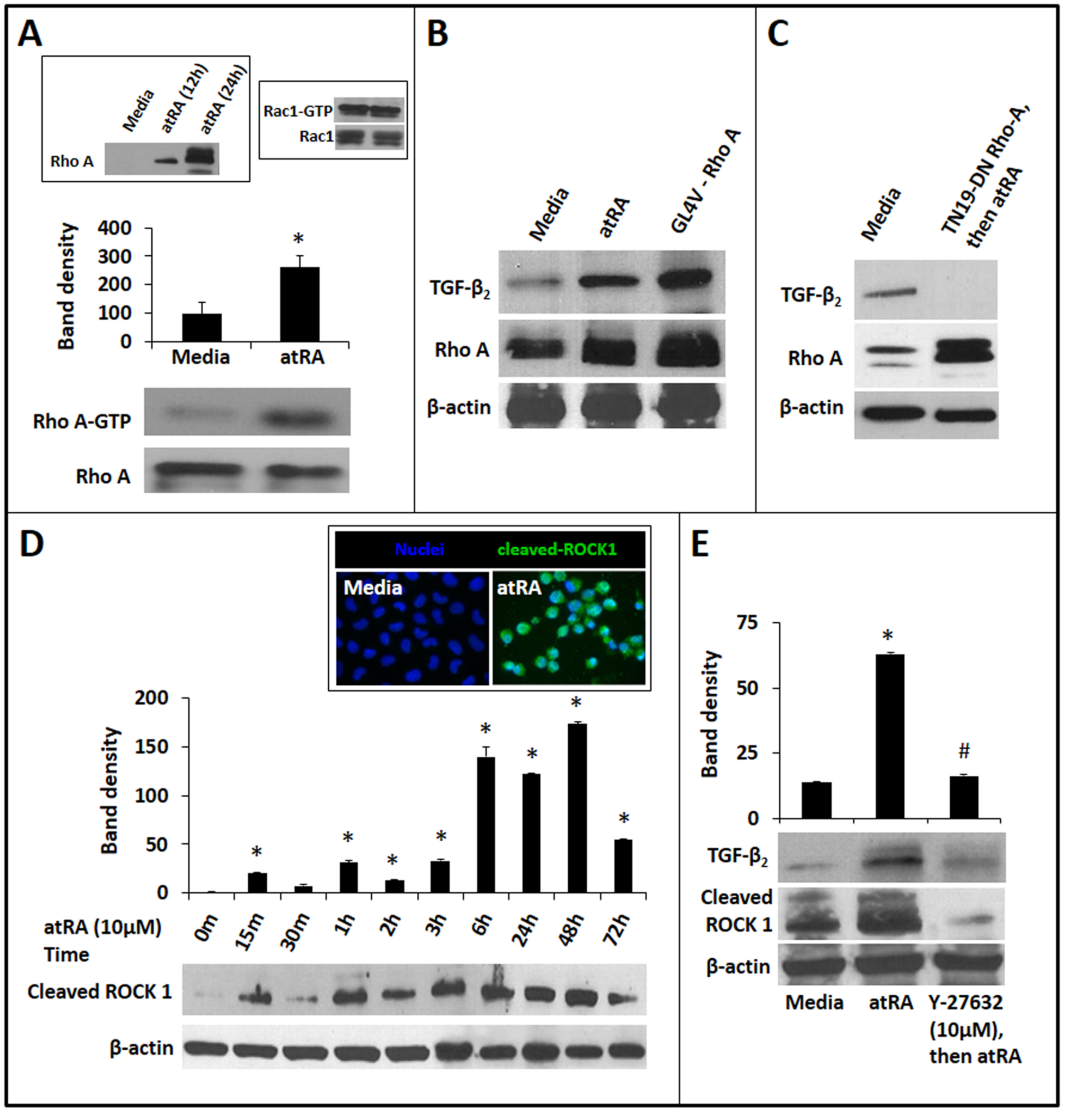
AtRA-induced TGF-β_2_ expression in IECs is mediated via RhoA GTPase and ROCK1. *A*. Representative Western blots show increased expression of activated RhoA (RhoA-GTP) in IEC6 cells treated with atRA × 4h. Activated RhoA was pulled down from cell lysates using Rhotekin-agarose beads. Bar-diagram (means ± SE) summarizes densitometric data. *Inset*: Left panel: ATRA also increased the expression of total RhoA in IECs. Right panel: AtRA-treatment did not increase Rac1-GTP in IEC6 cells. *B*. Western blots show that atRA-induced TGF-β_2_ expression in IEC6 cells was reproduced by over-expression of the constitutively-active GL4V mutant of RhoA. *C*. Cells expressing the TN19 dominant-negative RhoA mutant did not show atRA-induced TGF-β_2_ expression. *D*. Western blots show cleaved ROCK1 in IEC6 cells, depicted as a function of the duration of atRA treatment. Bar-diagram (means ± SE) summarizes densitometric data. *Inset*: Fluorescence photomicrographs (magnification 630x) show nuclear localization of ROCK1 (green) in IEC6 cells treated with atRA × 2h. Nuclear staining (blue) was obtained with DAPI (blue). *E*. Pharmacological inhibition of ROCK1 by Y-27632 blocked atRA-induced TGF-β_2_ expression in IEC6 cells. Western blots show TGF-β_2_ and cleaved ROCK1 expression. Bar-diagram (means ± SE) summarizes densitometric data, normalized against β-actin. Data represent 3 separate experiments; * *p*<0.05 compared to cells cultured in media alone; # indicates *p*<0.05 compared to atRA-treated cells.

Existing information indicates that atRA differentially activates the small GTPases RhoA, rac1, and cdc42 in different cell lineages [26–28]. To determine whether atRA activates one or more of these mediators in IECs, we used Rhotekin RBD- and PAK PBD-conjugated agarose beads to pull down the active forms of RhoA, rac1, and cdc42 from cell lysates and measured these GTPases in Western blots. As shown in **Fig 2A**, atRA increased active RhoA (RhoA-GTP) in IEC6 cells. There was no change in active Rac1, and we did not detect activated Cdc42 in atRA-treated cells. Consistent with these findings, IEC6 cells expressing the constitutively-active GL4V mutant of RhoA showed increased TGF-β_2_ expression similar to the effects of atRA (**Fig 2B**). In contrast, we did not find TGF-β_2_ expression in cells expressing the dominant-negative TN19 RhoA mutant, emphasizing the importance of RhoA in constitutive and atRA-induced TGF-β_2_ expression (**Fig 2C**).

To determine whether atRA activated RhoA-mediated signaling in IEC6 cells, we sought activation of downstream ROCK1 and therefore, measured the expression of cleaved ROCK1 by Western blots [29]. Cleaved ROCK1 was detected in atRA-treated cells within 15 min of treatment and lasted 72h with some oscillations (**Fig 2D**). In support of these data, we also detected increased immunoreactivity for cleaved ROCK1 in atRA-treated cells, where it was specifically localized in the cell nuclei (*inset*). Treatment of IEC6 cells with Y-27632, a selective inhibitor of ROCK activity, blocked atRA-induced TGF-β_2_ expression in these cells. Y-27632 also suppressed the expression of cleaved ROCK1, which is consistent with existing information indicating that ROCK1 activates caspase-3, cleaving ROCK1 and thereby setting up a feed-forward cycle [30]. We focused exclusively on ROCK1 and did not pursue ROCK2 because there was no consistent change in ROCK2 expression in atRA-treated cells (*not depicted*).

### AtRA-induced TGF-β_2_ expression in IECs is mediated via p38 MAPK

**Fig 3:**
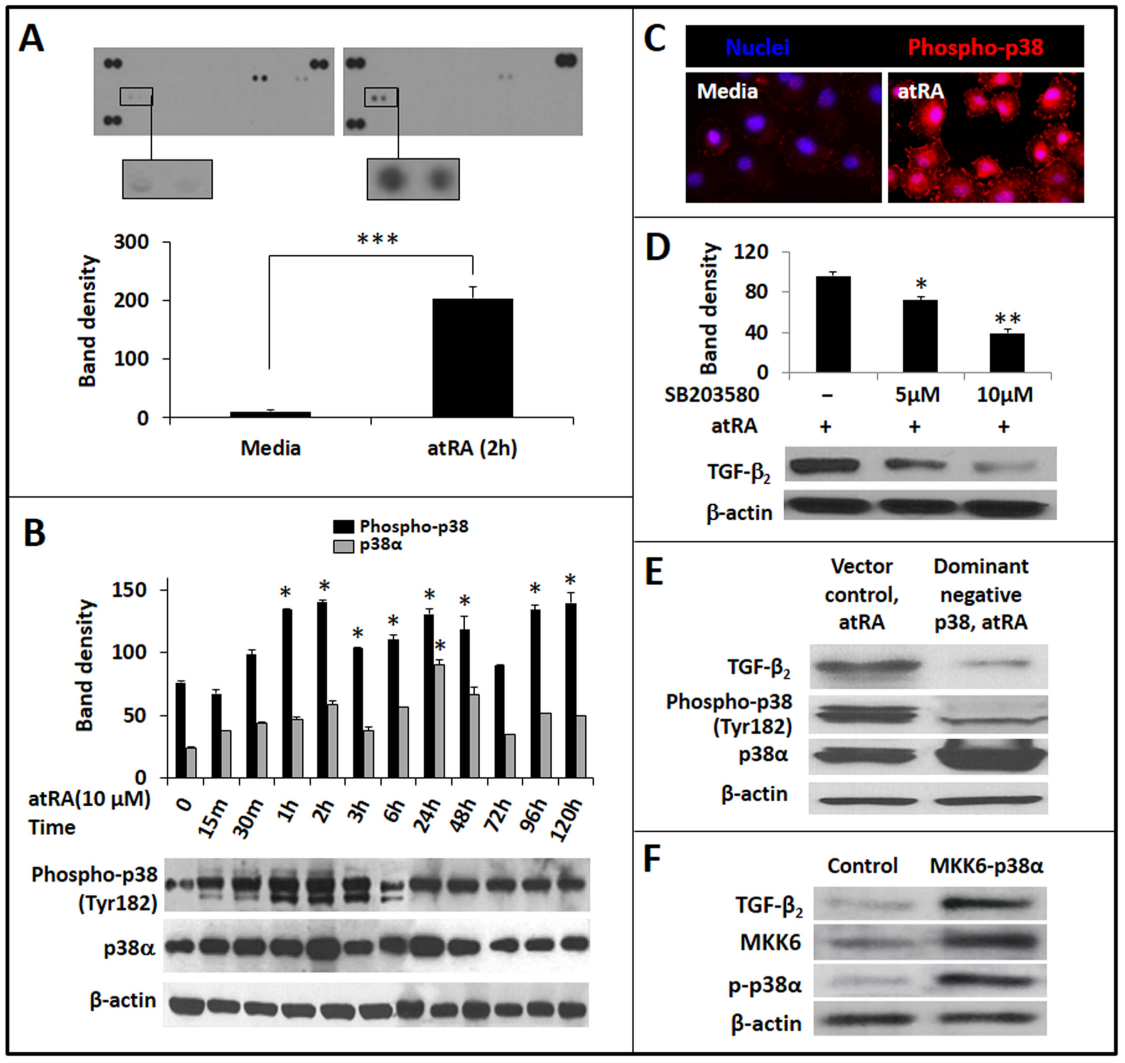
AtRA-induced TGF-β_2_ expression in IECs is mediated via p38 MAPK. *A*. Representative blots from a phospho-MAPK antibody array show increased phospho-p38α expression in IEC6 cells treated with atRA × 2h. Bar-diagram (means ± SE) summarizes densitometric data. *B*. Western blots show the effect of atRA on phospho-p38 (Tyr182), p38α, and β-actin expression in IEC6 cells, depicted as a function of the duration of atRA treatment. Bar-diagram (means ± SE) summarizes densitometric data for each analyte normalized against β-actin. *C*. Fluorescence photomicrographs (magnification 630x) show increased phospho-p38 immunoreactivity (red) in IEC6 cells treated with atRA × 2h. Nuclear staining (blue) was obtained with DAPI. *D*. Western blots show that pharmacological inhibition of p38 MAPK using SB203580 blocked atRA-induced TGF-β_2_ expression in IEC6 cells. Bar-diagram (means ± SE) summarizes densitometric data. *E*. IEC6 cells transduced to express a dominant-negative p38 transcript show decreased expression of TGF-β_2_ and phospho-p38 MAPK. We used the adenoviral vector in a multiplicity of infection of 40, which was determined to be the optimum dose in preliminary experiments. Bar-diagram (means ± SE) summarizes densitometric data. *F*. IEC6 cells transfected to express a MKK6-p38α fusion protein with constitutively-active p38α activity show increased TGF-β_2_ expression. Additional blots show increased MKK6 and phospho-p38 MAPK expression. Data represent 3 separate experiments; * *p*<0.05, ** *p*<0.01, *** *p*<0.001.

We next used a phospho-MAPK antibody array to identify MAPKs involved in atRA-induced TGF-β_2_ expression in IECs. Treatment with atRA significantly increased phosphorylated p38α. There was also a decrease in phospho-glycogen synthase kinase-3β and phospho-extracellular signal-regulated kinase-2 (**Fig 3A**). P38 activation has been previously shown to increase TGF-β_2_ transcription in human keloid fibroblasts [31], and therefore, we focused subsequent studies on this enzyme. We first confirmed these findings by measuring the temporal changes in phospho-p38 (Tyr182) expression in IEC6 cells following atRA treatment. As shown in **Fig 3B**, atRA caused a persistent increase in phospho-p38 expression starting at 1h. We also noted increased total p38α at 24h. Consistent with these findings, we also detected increased nuclear and cytoplasmic immunoreactivity of phospho-p38 following atRA treatment (**Fig 3C**). To determine the contribution of p38α to atRA-induced TGF-β_2_ expression, we used pharmacological and genetic approaches to block p38 activation in IEC6 cells. As shown in **Fig 3D**, SB203580, a specific inhibitor of p38, blocked atRA-induced TGF-β_2_ expression in a dose-dependent fashion. Similarly, cells transfected with a dominant-negative mutant of p38α failed to upregulate TGF-β_2_ in the presence of atRA (**Fig 3E**). The specific role of p38 in atRA-mediated TGF-β_2_ expression was confirmed in studies with pharmacological inhibitors of JNK, MEK/ERK, and the PI3K, which did not block TGF-β_2_ expression (*not depicted*). Finally, to confirm the central role of p38α in TGF-β_2_ expression, we transiently-transfected IEC6 cells to express a MKK6-p38α fusion protein that displays constitutively-active p38α activity. Consistent with the effects of atRA-mediated p38α activation, MKK6-p38α-expressing IEC6 cells also showed increased TGF-β_2_ expression (**Fig 3F**).

### AtRA-induced TGF-β_2_ expression in IECs is mediated via ATF2

**Fig 4:**
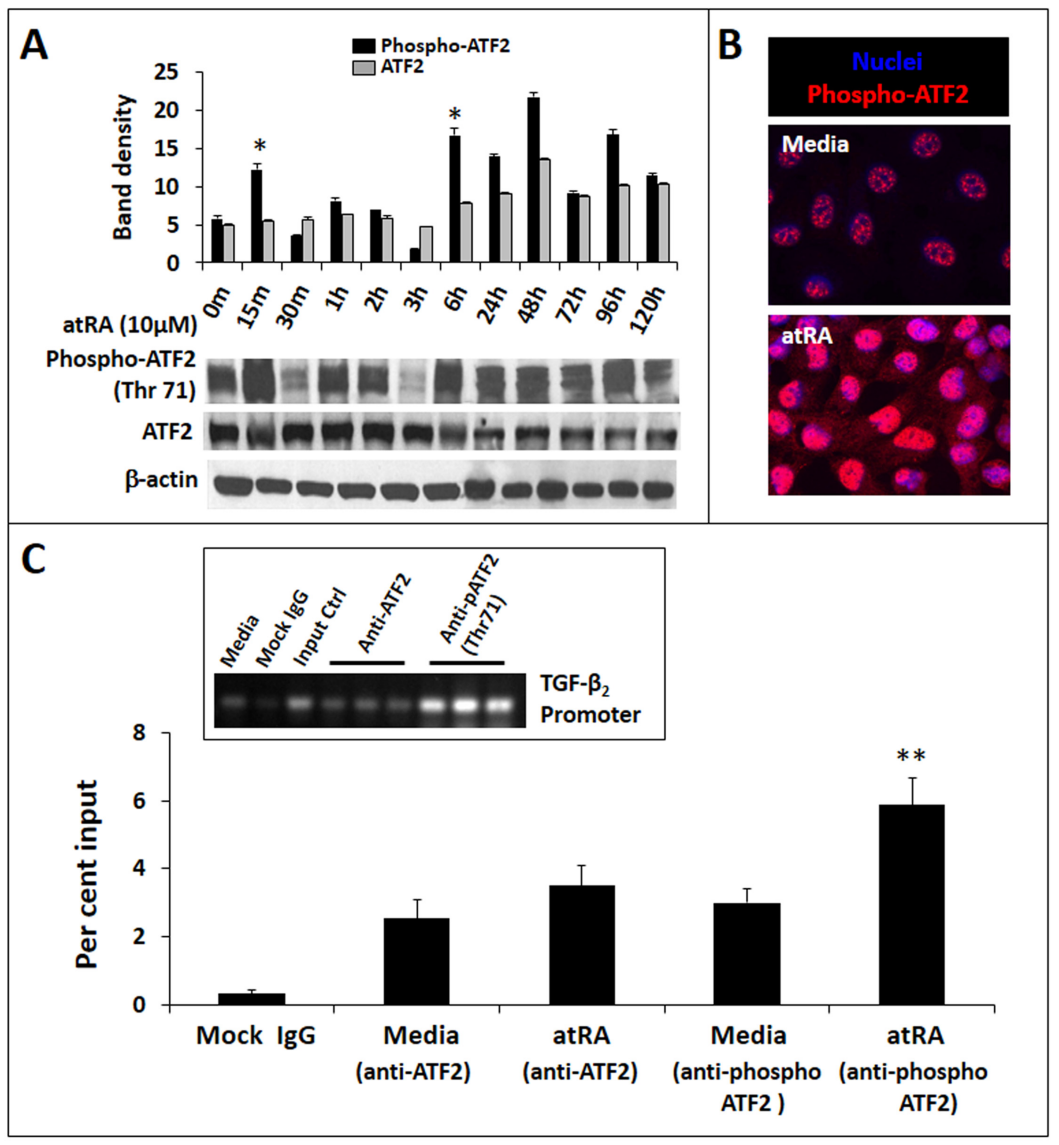
AtRA-induced TGF-β_2_ expression in IECs is mediated via ATF2. *A*. Western blots show phospho- and total ATF2 in IEC6 cells, depicted as a function of the duration of atRA treatment. Bar-diagram (means ± SE) summarizes densitometric data normalized against β-actin. *B*. Fluorescence photomicrographs (magnification 630x) show increased nuclear localization of phospho-ATF2 (red) in IEC6 cells treated with atRA × 2h. Nuclear staining (blue) was obtained with DAPI. *C*. AtRA treatment increases phospho-ATF2 binding to the TGF-β_2_ promoter. Bar diagram (means ± SE) shows data from chromatin immunoprecipitation (ChIP) assay of phospho-ATF2 binding to the TGF-β_2_ promoter region. Quantification of phospho-ATF2 binding was performed using real-time PCR and is shown as % input. To calculate % input, ChIP results for the specific antibody were determined using a standard curve of input DNA from the same cells. Control IgG ChIP results were subtracted from specific antibody ChIP results. *Inset*: Agarose gel showing enrichment of phospho-ATF2 on the TGF-β_2_ promoter. Data represent 3 separate experiments; * *p*<0.05, ** *p*<0.01.

Existing evidence indicates that the transcriptional factor ATF2, which is known to be activated by atRA [32], is a key regulator of TGF-β_2_ expression [33]. Therefore, we next asked whether atRA activates ATF2 in IECs. As shown in **Fig 4A**, treatment of IEC6 cells with atRA promoted ATF2 phosphorylation (Thr71), starting within 15 min and lasting for nearly 96h with some oscillations. In support of these findings, we also detected increased nuclear immunoreactivity of phospho-ATF2 in cultured IEC6 cells (**Fig 4B**). Phospho-ATF2 binding to the TGF-β_2_ promoter in atRA-treated cells was confirmed by ChIP (**Fig 4C**).

**Fig 5:**
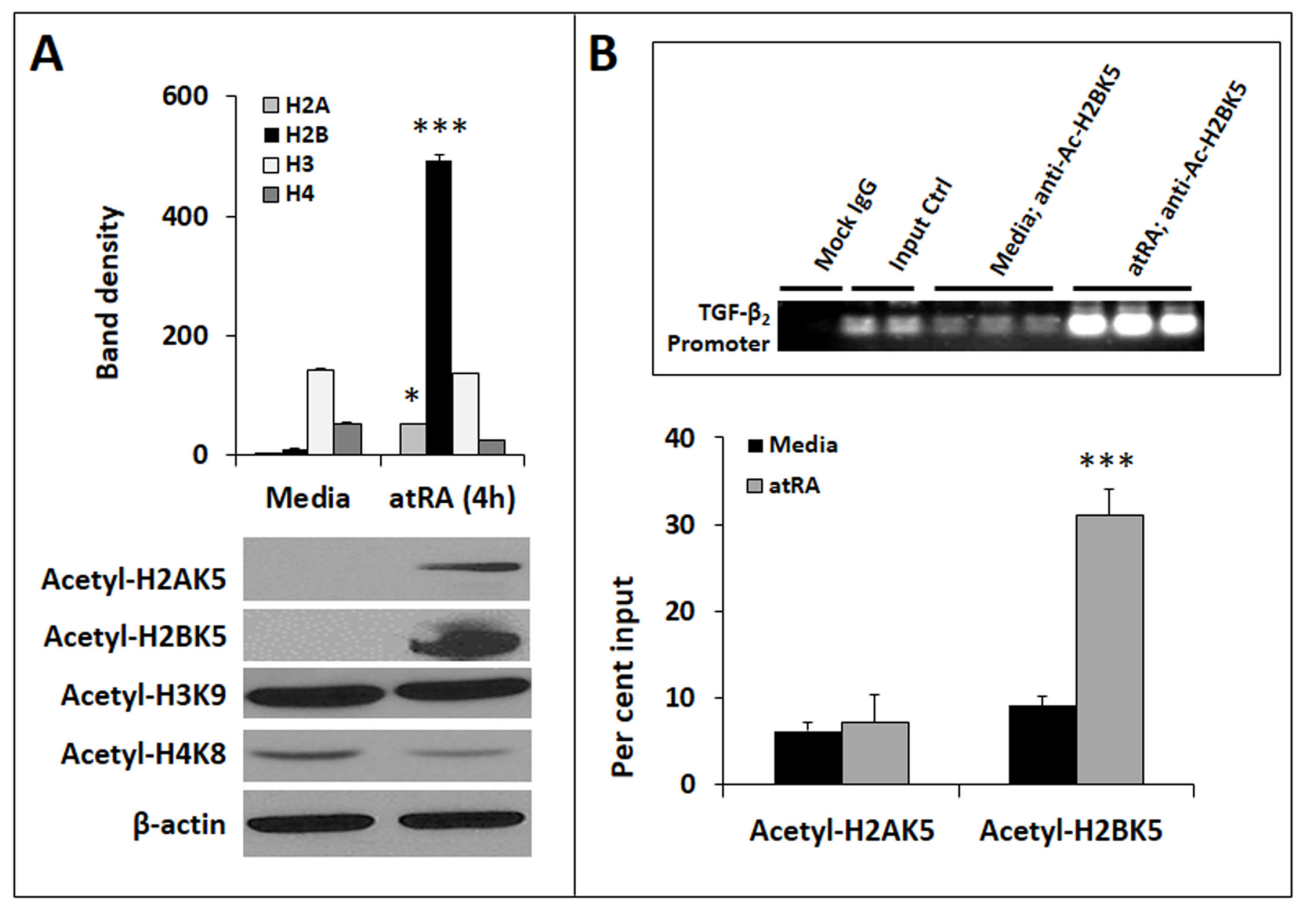
AtRA promotes the acetylation of histone H2B in the TGF-β_2_ nucleosome. ***A*.** Western blots show the genome-wide acetylation status of histones H2A, H2B, H3, and H4. Bar-diagram (means ± SE) summarizes densitometric data normalized against β-actin. ***B*.** AtRA treatment increases the acetylation of histone H2B (lys5) on the TGF-β_2_ nucleosome. Bar diagram (means ± SE) shows data from ChIP assay, where the acetylated histones were pulled down and the presence of the TGF-β_2_ promoter region in the complex confirmed by real-time PCR. Quantification of acetyl-H2B is shown as % input. *Inset*: Agarose gel showing enrichment of acetyl-H2B in the TGF-β_2_ promoter. Data represent 3 separate experiments; * *p*<0.05, *** *p*<0.001.

We have already shown that atRA increased TGF-β_2_ promoter activity (**Fig 1D**). To support these data, we asked whether treatment of IECs with atRA resulted in histone modifications typically associated with transcriptional activation [34]. Therefore, we sought acetyl-H2AK5, acetyl-H2BK5, acetyl-H3K9, and acetyl-H4K8 first in the entire chromatin and then specifically on the nucleosome of TGF-β_2._ In Western blots (**Fig 5A**), we detected a global increase in acetyl-H2AK5 and acetyl-H2BK5 in atRA-treated IEC6 cells. To confirm the presence of these modifications specifically on the TGF-β_2_ nucleosome, we treated IEC6 cells with atRA and performed ChIP, where nuclear extracts of formaldehyde-fixed cells were sonicated and then subjected to immunoprecipitation using anti-acetyl-H2AK5, anti-acetyl-H2BK5, or control IgG. As depicted in **Fig 5B**, quantitative PCR confirmed that atRA promoted H2BK5 acetylation on the TGF-β_2_ nucleosome in IEC6 cells.

### Both ROCK1 and p38α MAPK are required for atRA-mediated phosphorylation of ATF2 in IECs

**Fig 6:**
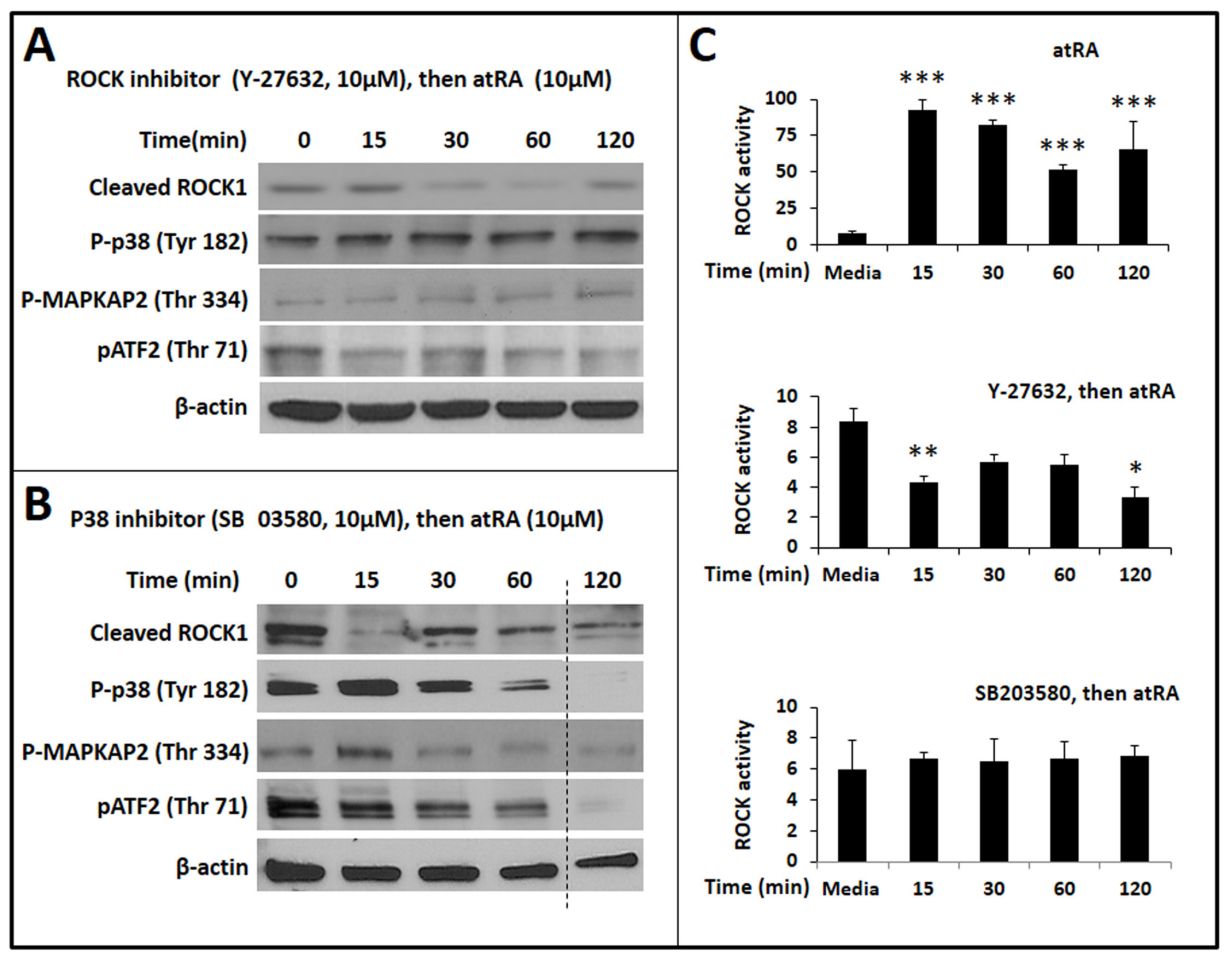
Both ROCK1 and p38α MAPK are required for atRA-mediated phosphorylation of ATF2 in IECs. ***A*.** Western blots show the effect of the ROCK inhibitor Y-27632 on the expression of cleaved ROCK1, phospho-p38, phospho-MAPKAPK2, and phospho-ATF2. Β-actin was used as the loading control. ***B*.** Effect of the p38 inhibitor SB203580 on the expression of cleaved ROCK1, phospho-p38, phospho-MAPKAPK2, and phospho-ATF2. Data represent 3 separate experiments. ***C*.** Bar-diagrams (means ± SE) summarize ROCK activity in IEC6 cells treated with atRA (10 μM, top panel), with Y-27632 (10 μM) followed by atRA (10 μM; middle panel), and SB203580 (10 μM) followed by atRA (10 μM; bottom panel). Data represent 3 separate experiments; * *p*<0.05, ** *p*<0.01, *** *p*<0.001.

To determine whether RhoA/ROCK1 and p38α signals are both required for atRA-mediated phosphorylation of ATF2 and TGF-β_2_ expression in IECs, we added Y-27632 and SB203580 to inhibit ROCK and p38 activity, respectively, and measured cleaved ROCK1, phospho-p38, phospho-MAPKAPK2, and phospho-ATF2. Y-27632 suppressed atRA-induced expression of the cleaved ROCK1 fragment, phosphorylation of p38 MAPK and its downstream MAPKAPK2, and phosphorylation of ATF2 (**Fig 6A**). Similarly, SB203580 blocked atRA-mediated ATF2 phosphorylation (**Fig 6B**). Interestingly, SB203580 inhibited atRA-induced expression of cleaved ROCK1, indicating that p38α contributes to atRA-mediated activation of ROCK1. These findings were confirmed in IEC6 cells expressing the DN variant of p38. The inhibitory effect of SB203580 on atRA-activated RhoA signaling was confirmed in direct measurements of ROCK activity in IEC6 cells (**Fig 6C**).

### AtRA modulates Smad expression in IECs

**Fig 7:**
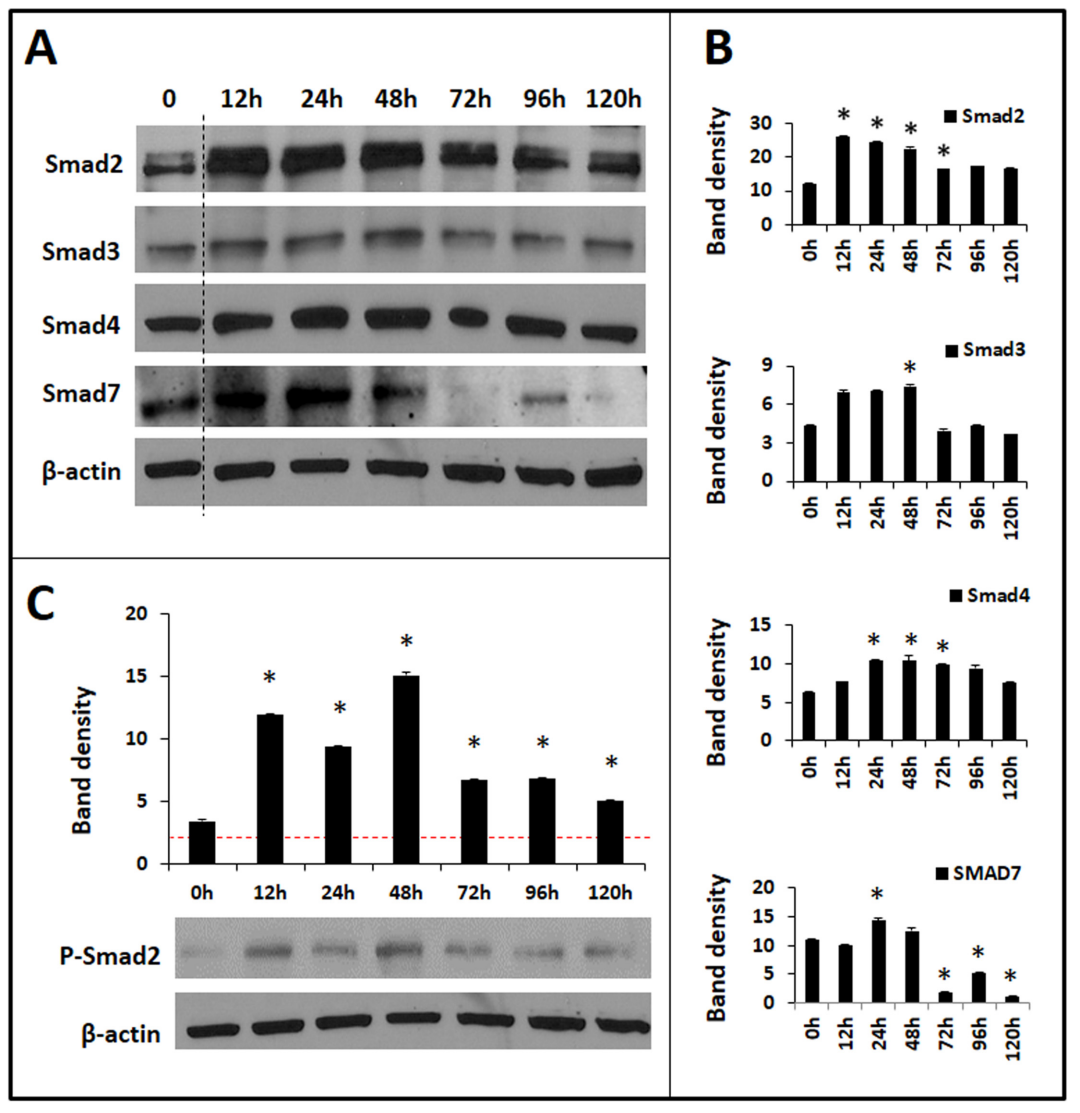
AtRA modulates Smad expression in IECs. ***A*.** Western blots show the expression of Smad2, Smad3, Smad4, and Smad7 in atRA-treated IEC6 cells, shown as a function of time. ***B*.** Bar-diagrams (means ± SE) summarize densitometric data of blots in panel A, normalized against β-actin. ***C*.** Western blots show phospho-Smad2 expression as a function of time after atRA treatment. Data represent 3 separate experiments; * *p*<0.05.

We previously showed that neonatal IECs constitutively express Smad7, which dampens the normal autocrine induction of TGF-β_2_ in these cells [2]. To investigate how atRA circumvents the inhibitory effects of Smad7, we examined atRA effects on Smad2, Smad3, Smad4, and Smad7, the 4 key Smads expressed in the developing intestine [35]. AtRA induced the expression of all the 4 Smads in IEC6 cells, but major differences were noted in the temporal kinetics of such induction (**Fig 7A and 7B**). AtRA-treated cells showed a persistent increase in Smad2 for nearly 96h, whereas Smad7 expression first rose at 12–24h and then decreased below basal levels. Therefore, at delayed time-points, the activating Smads (Smad2, Smad3) were still expressed at high levels whereas Smad7 was not. This bias towards activating Smads was evident in atRA-treated cells as a persistent increase in phospho-Smad2 expression, an indicator of ongoing TGF-β signaling, up to 96h (**Fig 7C**).

## Discussion

We present a detailed investigation into the effects of atRA on TGF-β_2_ expression in IECs. We show that pharmacological doses of atRA can induce TGF-β_2_ expression in IEC6 cells, and that these effects were mediated via the RhoA GTPase, ROCK1, p38α MAPK, and ATF2, which activated the TGF-β_2_ promoter. To our knowledge, this is the first study to elucidate the signaling mechanisms for atRA-induced TGF-β_2_ expression in intestinal cells. TGF-β_2_ promotes barrier function, immune tolerance, and mucosal restitution in the developing intestine [36–39], and may protect against NEC and allergic disorders. In this context, these findings raise interesting translational possibilities that merit further investigation in preclinical and clinical settings.

AtRA is the carboxylic acid form of vitamin A (all-*trans* retinol) and is a well-acknowledged signaling mediator during gestational development with a wide spectrum of biological activities, such as cell differentiation, morphogenesis, and epithelial-mesenchymal interaction in the developing intestine, kidney, lung, and the central nervous system [40–42]. In our study, atRA stimulated a rapid and persistent increase in TGF-β_2_ mRNA and protein in IECs. These findings are consistent with previous reports in many other epithelial lineages in skin and associated appendages, and in lung and pancreatic cancer cells [9–12]. AtRA effects are receptor-mediated, and in IECs, it can induce the three retinoic acid receptor (RAR) isoforms RAR-α, RAR-β, and RAR-γ [43]. During fetal development, the importance of endogenous retinoic acid in the regulation of TGF-β_2_ expression is evident from the phenotypic similarities between TGF-β_2_-null mice with the offspring of vitamin A-deficient mice and the retinoic acid receptor-αγ and -βγ compound null animals [44–48]. However, further study is needed to elucidate the role of endogenous retinoic acid in mucosal homeostasis in the preterm intestine and the risk of NEC in these infants. The spatiotemporal variability in retinoic acid effects in fetal tissues [13–16] can sometimes be explained on the basis of differences in the expression of the retinoic acid receptors [49], but more comprehensive genome-wide approaches are needed to truly understand the complex gene expression networks activated by retinoic acid [50,51].

In our study, atRA activated RhoA in IEC6 cells. Existing studies indicate that retinoic acid can differentially activate various Rho family GTPases in various cell lineages [26–28]. In neuronal cells, atRA activated tissue transglutaminase, which resulted in transamidation and persistent activation of RhoA and downstream ROCK2 [28]. These observations contrast with our findings in IECs, where atRA increased the GTP-bound form of RhoA, and that RhoA was required for both constitutive and atRA-induced TGF-β_2_ expression. In IECs, atRA-induced RhoA activation was associated with the appearance of the constitutively-active, cleaved fragment of ROCK1. Pharmacological inhibitors of ROCK blocked atRA-induced TGF-β_2_ expression, emphasizing the role of ROCK1 in this process. The oscillations we observed in cleaved RhoA expression (and also in ATF2 phosphorylation) are widely recognized in signaling cascades, and may indicate the presence of negative feedback loops, competing multisite phosphorylation, or sequestration of the target protein by enzyme(s) [52,53]. Although the physiological relevance of this oscillatory dynamics of biochemical systems is not well-understood, it could play a role in maintaining cellular sensitivity to persistent signaling events [54].

In IECs, atRA-induced TGF-β_2_ expression was mediated via activation of the p38α MAPK. Our findings are consistent with the observations of Fernández-Calotti *et al*. [55], who showed atRA induced TGF-β_1_ expression in chronic lymphoblastic leukemia cells via a RhoA-p38 mediated pathway. In another study, Alsayed *et al*. [26] demonstrated that atRA activated the p38 MAPK in leukemic and breast cancer cell lines. In these cells, atRA also activated the rac1 GTPase, which was essential for the activation of p38. However, the authors concluded that atRA-dependent p38 activation did not increase the transcriptional activation of downstream genes such as stat1, which contrasts with our findings where atRA-induced TGF-β_2_ expression was blocked by pharmacological/genetic inhibitors of p38. In other cell lineages, atRA can also interact with other MAPKs; atRA-mediated ERK2 activation promoted cell differentiation in HL-60 acute myelogenous leukemia cells [56], and blocked JNK kinase-dependent signaling pathways [57] by activating MAP kinase phosphatase-1 and inhibiting MKK4 [58].

The ATF2 transcription factor belongs to the bZip (*basic leucine zipper* domain) superfamily of DNA-binding proteins. ATF2 homodimers bind cAMP-responsive element (CRE)-like sequences [59,60], including to the one present on the TGF-β_2_ promoter [33]. ATF2 carries a nuclear export signal in its leucine zipper region and two nuclear localization signals in its basic region, and normally shuttles between the cytoplasm and the nucleus. In the nucleus, ATF2 is activated by MAPKs such as p38, which phosphorylate its Thr69, Thr71, and Ser91 residues in the *N*-terminal activation domain [61]. In the present study, we have shown atRA-mediated ATF2 phosphorylation (Thr71), phospho-ATF2 binding to the TGF-β_2_ promoter, increased promoter activity of the TGF-β_2_ promoter, and the development of histone modifications on the TGF-β_2_ nucleosome that are typically associated with chromatin decondensation and transcriptional activation [34]. These findings emphasize transcriptional activation as an important mechanism for the upregulation of TGF-β_2_ in atRA-treated IECs. The histone modifications we noted are consistent with existing information indicating that phosphorylated ATF2 acquires intrinsic histone acetyltransferase activity [62] and can promote H2B acetylation in other cell lineages [63]. AtRA produced a fairly persistent increase in TGF-β_2_ expression, which could be traced to the persistent increase in activating Smads and its likely effects on the autocrine induction of TGF-β_2_ [2]. However, further study is needed to exclude the effects of atRA on the stability of TGF-β_2_ transcripts [9].

In conclusion, we show that atRA can stimulate TGF-β_2_ expression in IECs, which may have important implications in the development of new preventive/therapeutic strategies against NEC and gastrointestinal allergies. Although atRA and vitamin A effects on intestinal injury have been investigated previously, existing studies were based on the presumed ‘global’ effects of these agents on epithelial maturation. Because these studies were not based on specific, measureable effects of atRA, there was wide variability in doses and outcome measures. Ozdemir *et al*. [64] showed that intraperitoneal administration of atRA may attenuate NEC-like inflammatory intestinal injury in rat pups. In preliminary studies, we have also noted a similar protective effect of orally-administered atRA in intestinal injury induced in mouse pups by intraperitoneal administration of platelet-activating factor and lipopolysaccharide [65]. Yuen and Stratford [66] noted improvement in tissue healing when vitamin A was added to *ex* planted intestinal tissue from piglets with NEC-like injury. In another study, Nafday *et al*. [67] showed that vitamin A supplementation ameliorates butyric acid-induced colonic injury in rats. The protective effects of vitamin A were not evident in clinical studies focused on prevention of chronic lung disease [68,69], but these studies were not powered to investigate the effects on NEC. Findings from the present study may facilitate the development of optimum dosing regimens and improved study design focused on maximizing gut mucosal TGF-β_2_ expression and its effects on intestinal inflammation.
